# Aqua­{4,4′,6,6′-tetra­fluoro-2,2′-[(piperazine-1,4-di­yl)dimethyl­ene]diphenolato}copper(II)

**DOI:** 10.1107/S1600536810040080

**Published:** 2010-10-13

**Authors:** Koji Kubono, Yuki Tsuno, Keita Tani, Kunihiko Yokoi

**Affiliations:** a Division of Natural Sciences, Osaka Kyoiku University, Kashiwara, Osaka 582-8582, Japan

## Abstract

In the title compound, [Cu(C_18_H_16_F_4_N_2_O_2_)(H_2_O)], the Cu^II^ atom shows a distorted square-pyramidal coordination geometry with the *N*,*N*′,*O*,*O*′-tetra­dentate piperazine–diphenolate ligand forming the basal plane. The apical site is occupied by the O atom of a coordinated water mol­ecule. Neighbouring complexes are associated through inter­molecular O—H⋯O and O—H⋯F hydrogen bonds between the water mol­ecule and a phenolate O atom or an F atom from an adjacent ligand, respectively, forming a centrosymmetric dimer. Dimers are linked by additional inter­molecular C—H⋯O and C—H⋯F hydrogen bonds, giving infinite chains propagating along the *a* axis.

## Related literature

For related stuctures, see: Kubono *et al.* (2003 ▶, 2009 ▶); Loukiala *et al.* (1997 ▶); Mukhopadhyay *et al.* (2004 ▶); Weinberger *et al.* (2000 ▶). For the supra­molecular chemistry of complexes with piperazine-based ligands, see: Tsai *et al.* (2008 ▶); Zhao *et al.* (2004 ▶). For graph-set analysis in the crystal structures of organometallic compounds, see: Bernstein *et al.* (1995 ▶).
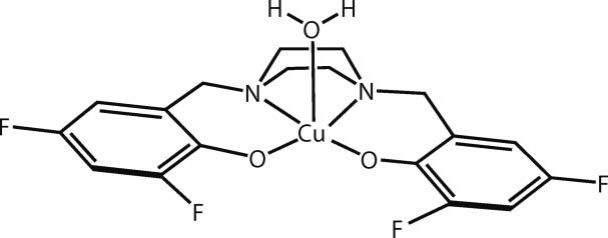


## Experimental

### 

#### Crystal data

[Cu(C_18_H_16_F_4_N_2_O_2_)(H_2_O)]
*M* *_r_* = 449.89
Triclinic, 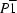

*a* = 8.0157 (17) Å
*b* = 9.6873 (10) Å
*c* = 11.7693 (12) Å
α = 83.743 (9)°
β = 87.763 (12)°
γ = 74.420 (11)°
*V* = 875.0 (2) Å^3^
*Z* = 2
Mo *K*α radiation
μ = 1.31 mm^−1^
*T* = 296 K
0.30 × 0.20 × 0.10 mm

#### Data collection

Rigaku AFC-7R diffractometer
Absorption correction: ψ scan (North *et al.*, 1968 ▶) *T*_min_ = 0.737, *T*_max_ = 0.877
4895 measured reflections
4015 independent reflections
3120 reflections with *I* > 2σ(*I*)
*R*_int_ = 0.051
3 standard reflections every 150 reflections  intensity decay: 1.1%

#### Refinement

*R*[*F* ^2^ > 2σ(*F* ^2^)] = 0.039
*wR*(*F* ^2^) = 0.108
*S* = 1.05
4015 reflections
262 parameters
H atoms treated by a mixture of independent and constrained refinement
Δρ_max_ = 0.54 e Å^−3^
Δρ_min_ = −0.61 e Å^−3^

### 

Data collection: *WinAFC* (Rigaku/MSC, 2006 ▶); cell refinement: *WinAFC*; data reduction: *CrystalStructure* (Rigaku/MSC, 2006 ▶); program(s) used to solve structure: *SIR92* (Altomare *et al.*, 1993 ▶); program(s) used to refine structure: *SHELXL97* (Sheldrick, 2008 ▶); molecular graphics: *PLATON* (Spek, 2009 ▶); software used to prepare material for publication: *CrystalStructure*.

## Supplementary Material

Crystal structure: contains datablocks global, I. DOI: 10.1107/S1600536810040080/im2234sup1.cif

Structure factors: contains datablocks I. DOI: 10.1107/S1600536810040080/im2234Isup2.hkl

Additional supplementary materials:  crystallographic information; 3D view; checkCIF report

## Figures and Tables

**Table 1:** Hydrogen-bond geometry (Å, °)

*D*—H⋯*A*	*D*—H	H⋯*A*	*D*⋯*A*	*D*—H⋯*A*
O3—H17⋯O2^i^	0.77 (5)	2.14 (5)	2.852 (4)	154 (5)
O3—H18⋯F1^i^	0.76 (6)	2.41 (6)	3.122 (3)	156 (6)
C7—H3⋯O2^ii^	0.97	2.49	3.376 (3)	152 (1)
C11—H12⋯O1^ii^	0.97	2.50	3.226 (3)	132 (1)
C8—H6⋯F4^iii^	0.97	2.54	3.356 (4)	143 (1)
C12—H13⋯F1^i^	0.97	2.32	3.117 (4)	140 (1)

Symmetry codes: (i) 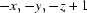; (ii) 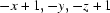; (iii) 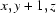.

**Fig. 1.**
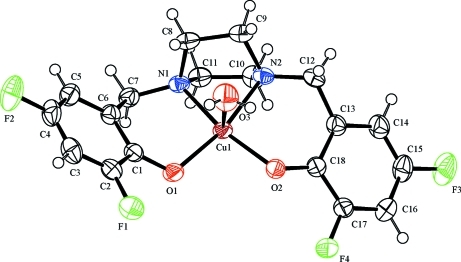
The molecule of the title complex showing the atom-labelling scheme. Displacement ellipsoids are drawn at the 50% probability level and H atoms are represented by circles of arbitrary size.

**Fig. 2.**
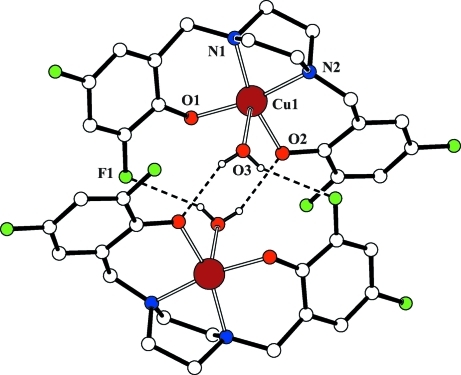
Dimeric structure of the title complex, with the hydrogen atoms bound to carbon being omitted for clarity. The O—H···O and O—H···F hydrogen bonds are shown as dashed lines.

**Fig. 3.**
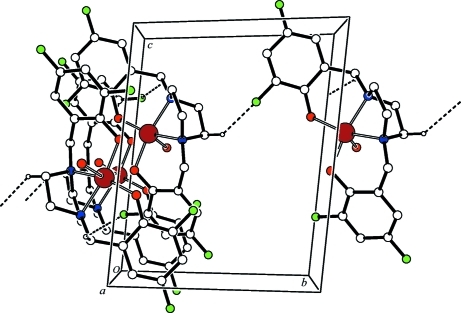
Packing diagram of the title complex, viewed down the a axis. The C—H···F hydrogen bonds are shown as dashed lines.

## supplementary crystallographic information

### Comment

Piperazine adopts chair and boat conformations by complexing various metal ions, showing several coordination modes and geometries (Kubono *et al.*, 2003; Loukiala *et al.* 1997; Mukhopadhyay *et al.* 2004; Weinberger *et al.* 2000). Therefore, metal complexes with piperazine based ligands have been of great interest in coordination and supramolecular chemistry (Tsai *et al.*, 2008; Zhao *et al.*, 2004). Recently, we have reported the crystal structure of a Cu^II^ complex with tetrachloro-2,2'-(piperazine-1,4-diyldimethylene)diphenolate, Cu_2_(Cl_2_bpi)_2_, (Kubono *et al.*, 2009), which is a centrosymmetric dinuclear complex. As a continuation of this work on the structural characterization of piperazine-diphenolato compounds, the title mononuclear Cu(II) complex with difluorophenol derivative of the Cl_2_bpi ligand is reported here (Fig. 1).

The Cu(II) atom shows a distorted square-pyramidal coordination geometry with the basal plane comprised of two phenolate O and two tertiary alkyl N atoms from a piperazine-diphenolato ligand. The apical site is occupied by the O atom of a water molecule. The orientaion of two benzene rings in the title complex is anti-parallel, different from that in the dichlorophenol derivative, Cu_2_(Cl_2_bpi)_2_ (Kubono *et al.*, 2009). The difference is reflected in the torsion angles C10—N2—C12—C13 [-69.9 (3) ° in the title complex and -171.8 (4) ° in Cu_2_(Cl_2_bpi)_2_]. Bond lengths and angles involving copper are comparable to those observed in related complexes (Kubono *et al.*, 2009; Loukiala *et al.* 1997; Mukhopadhyay *et al.* 2004; Weinberger *et al.* 2000).

Neighbouring mononuclear complexs are associated through O—H···O and O—H···F intermolecular hydrogen bonds between the H atoms in the water ligand and a phenolate O atom or a F atom from an adjacent ligand generated by inversion operation, forming a centrosymmetric dimer (Fig. 2). The dimeric structure of the title complex is different from those of Cu_2_(Cl_2_bpi)_2_ and the dimethylphenolato derivative (Mukhopadhyay *et al.*, 2004), which are *µ*-type complexes bridged by a phenolate O atom from an adjacent ligand. The Cu1···Cu1^i^ distance within the dimer of the title compound is 5.5646 (6) Å [symmetry code: (i) -*x*, -*y*, -*z* + 1.]. The dimeric structure of the complex is additionally stabilized by intermolecular C12—H13···F1^i^ hydrogen bonds (Table 1).

In the crystal structure of the title complex, there are intermolecular C—H···O hydrogen bonds (Table 1), connecting the dimers. C7—H3···O2^ii^ [symmetry code: (ii) -*x* + 1, -*y*, -*z* + 1.] and C12—H13···F1^i^ hydrogen bonds form an infinite chain of the *C*(11) type (Bernstein *et al.*, 1995) propagating parallel to the *a* axis. Chains of dimers are crosslinked into a three-dimensional framework by C8—H6···F4^iii^ hydrogen bonds [symmetry code: (iii) *x*, *y* + 1, *z*.] (Fig. 3).

### Experimental

The ligand, H_2_F_2_bpi, was prepared by heating 2,4-difluorophenol (190 mmol), piperazine (95 mmol) and paraformaldehyde (190 mmol) under reflux in methanol for 6 h. The mixture was cooled to room temperature, then the solvent was evaporated under vacuum. The product was recrystallized from chloroform-methanol to give colorless ligand crystals (yield 36%). H_2_F_2_bpi (0.1 mmol) was dissolved in 60 ml hot methanol. Then 1 ml of a aqueous solution of copper acetate monohydrate (0.15 mmol) was added to this solution. The mixture was stirred for 30 min at 340 K. After a few weeks at room temperature, green crystals of (I) were obtained (yield 30%). Analysis calculated for C_18_H_18_CuF_4_N_2_O_3_: C 48.05, H 4.03, N 6.23%; found: C 47.92, H 4.08, N 6.18%.

### Refinement

All H atoms bound to carbon were placed at idealized positions and refined using a riding model, with C—H = 0.93–0.97Å and *U*_iso_(H) = 1.2 *U*_eq_(C). H atoms bound to the water O atom were found in a difference Fourier map, and then refined isotropically.

### Crystal data

**Table:**

[Cu(C_18_H_16_F_4_N_2_O_2_)(H_2_O)]	*Z* = 2
*M**_r_* = 449.89	*F*(000) = 458.00
Triclinic, *P*1	*D*_x_ = 1.708 Mg m^−^^3^
Hall symbol: -P 1	Mo *K*α radiation, λ = 0.71069 Å
*a* = 8.0157 (17) Å	Cell parameters from 25 reflections
*b* = 9.6873 (10) Å	θ = 15.2–16.8°
*c* = 11.7693 (12) Å	µ = 1.31 mm^−^^1^
α = 83.743 (9)°	*T* = 296 K
β = 87.763 (12)°	Prismatic, blue
γ = 74.420 (11)°	0.30 × 0.20 × 0.10 mm
*V* = 875.0 (2) Å^3^	

### Data collection

**Table:**

Rigaku AFC-7R diffractometer	*R*_int_ = 0.051
ω–2θ scans	θ_max_ = 27.5°
Absorption correction: ψ scan (North *et al.*, 1968)	*h* = −10→5
*T*_min_ = 0.737, *T*_max_ = 0.877	*k* = −12→12
4895 measured reflections	*l* = −15→15
4015 independent reflections	3 standard reflections every 150 reflections
3120 reflections with *F*^2^ > 2σ(*F*^2^)	intensity decay: 1.1%

### Refinement

**Table:**

Refinement on *F*^2^	H atoms treated by a mixture of independent and constrained refinement
*R*[*F*^2^ > 2σ(*F*^2^)] = 0.039	*w* = 1/[σ^2^(*F*_o_^2^) + (0.0368*P*)^2^ + 1.0373*P*] where *P* = (*F*_o_^2^ + 2*F*_c_^2^)/3
*wR*(*F*^2^) = 0.108	(Δ/σ)_max_ < 0.001
*S* = 1.05	Δρ_max_ = 0.54 e Å^−^^3^
4015 reflections	Δρ_min_ = −0.61 e Å^−^^3^
262 parameters	

### Special details

**Table:**

Geometry. All e.s.d.'s (except the e.s.d. in the dihedral angle between two l.s. planes) are estimated using the full covariance matrix. The cell e.s.d.'s are taken into account individually in the estimation of e.s.d.'s in distances, angles and torsion angles; correlations between e.s.d.'s in cell parameters are only used when they are defined by crystal symmetry. An approximate (isotropic) treatment of cell e.s.d.'s is used for estimating e.s.d.'s involving l.s. planes.
Refinement. Refinement of *F*^2^ against ALL reflections. The weighted *R*-factor *wR* and goodness of fit *S* are based on *F*^2^, conventional *R*-factors *R* are based on *F*, with *F* set to zero for negative *F*^2^. The threshold expression of *F*^2^ > σ(*F*^2^) is used only for calculating *R*-factors(gt) *etc*. and is not relevant to the choice of reflections for refinement. *R*-factors based on *F*^2^ are statistically about twice as large as those based on *F*, and *R*- factors based on ALL data will be even larger.

### Fractional atomic coordinates and isotropic or equivalent isotropic displacement parameters (Å^2^)

**Table:**

	*x*	*y*	*z*	*U*_iso_*/*U*_eq_	
Cu1	0.26597 (5)	0.08830 (4)	0.58962 (3)	0.03020 (12)	
F1	0.1709 (2)	−0.0061 (2)	0.24903 (17)	0.0463 (4)	
F2	0.1403 (3)	0.4645 (2)	0.0841 (2)	0.0726 (7)	
F3	0.3363 (3)	−0.3057 (2)	1.10318 (18)	0.0675 (7)	
F4	0.3025 (3)	−0.3709 (2)	0.71553 (17)	0.0510 (5)	
O1	0.3198 (3)	0.0354 (2)	0.43752 (17)	0.0367 (4)	
O2	0.2095 (3)	−0.0851 (2)	0.65512 (17)	0.0352 (4)	
O3	−0.0692 (4)	0.1793 (3)	0.5321 (2)	0.0542 (7)	
N1	0.3452 (3)	0.2713 (2)	0.5630 (2)	0.0296 (5)	
N2	0.2158 (3)	0.1861 (2)	0.7367 (2)	0.0302 (5)	
C1	0.2816 (3)	0.1421 (3)	0.3534 (2)	0.0311 (6)	
C2	0.2011 (4)	0.1248 (3)	0.2551 (2)	0.0352 (6)	
C3	0.1514 (4)	0.2286 (4)	0.1652 (2)	0.0439 (7)	
C4	0.1890 (4)	0.3584 (4)	0.1722 (2)	0.0456 (8)	
C5	0.2728 (4)	0.3836 (3)	0.2633 (2)	0.0398 (7)	
C6	0.3215 (4)	0.2762 (3)	0.3542 (2)	0.0335 (6)	
C7	0.4255 (4)	0.2979 (3)	0.4507 (2)	0.0337 (6)	
C8	0.1833 (4)	0.3809 (3)	0.5837 (2)	0.0368 (6)	
C9	0.1110 (4)	0.3332 (3)	0.6997 (2)	0.0374 (6)	
C10	0.3909 (4)	0.1940 (3)	0.7660 (2)	0.0385 (7)	
C11	0.4658 (4)	0.2616 (3)	0.6585 (2)	0.0347 (6)	
C12	0.1324 (4)	0.1160 (3)	0.8309 (2)	0.0369 (6)	
C13	0.2144 (4)	−0.0436 (3)	0.8547 (2)	0.0344 (6)	
C14	0.2497 (4)	−0.1032 (3)	0.9671 (2)	0.0423 (7)	
C15	0.3025 (5)	−0.2496 (4)	0.9919 (2)	0.0450 (8)	
C16	0.3221 (4)	−0.3418 (3)	0.9091 (3)	0.0429 (7)	
C17	0.2888 (4)	−0.2811 (3)	0.7984 (2)	0.0358 (6)	
C18	0.2376 (4)	−0.1328 (3)	0.7648 (2)	0.0318 (6)	
H1	0.0950	0.2124	0.1024	0.053*	
H2	0.2974	0.4718	0.2649	0.048*	
H3	0.5409	0.2332	0.4482	0.040*	
H4	0.4364	0.3958	0.4413	0.040*	
H5	0.1010	0.3881	0.5237	0.044*	
H6	0.2057	0.4742	0.5853	0.044*	
H7	0.1181	0.3983	0.7553	0.045*	
H8	−0.0094	0.3341	0.6925	0.045*	
H9	0.4641	0.0984	0.7889	0.046*	
H10	0.3838	0.2527	0.8286	0.046*	
H11	0.4753	0.3568	0.6704	0.042*	
H12	0.5802	0.2023	0.6410	0.042*	
H13	0.0113	0.1312	0.8128	0.044*	
H14	0.1370	0.1617	0.8997	0.044*	
H15	0.2374	−0.0434	1.0253	0.051*	
H16	0.3564	−0.4412	0.9270	0.052*	
H17	−0.108 (6)	0.180 (5)	0.473 (4)	0.064 (14)*	
H18	−0.093 (8)	0.120 (6)	0.572 (5)	0.10 (2)*	

### Atomic displacement parameters (Å^2^)

**Table:**

	*U*^11^	*U*^22^	*U*^33^	*U*^12^	*U*^13^	*U*^23^
Cu1	0.0424 (2)	0.02358 (18)	0.02562 (18)	−0.00841 (14)	−0.00367 (14)	−0.00654 (12)
F1	0.0546 (12)	0.0506 (11)	0.0397 (10)	−0.0196 (9)	−0.0076 (8)	−0.0136 (8)
F2	0.0874 (18)	0.0779 (16)	0.0472 (13)	−0.0242 (14)	−0.0234 (12)	0.0307 (11)
F3	0.0991 (19)	0.0711 (15)	0.0371 (11)	−0.0354 (14)	−0.0242 (12)	0.0138 (10)
F4	0.0811 (15)	0.0334 (9)	0.0425 (10)	−0.0203 (10)	0.0101 (10)	−0.0120 (8)
O1	0.0559 (14)	0.0271 (10)	0.0261 (10)	−0.0075 (9)	−0.0072 (9)	−0.0048 (7)
O2	0.0538 (13)	0.0314 (10)	0.0254 (9)	−0.0181 (9)	−0.0004 (9)	−0.0077 (8)
O3	0.0628 (18)	0.0647 (18)	0.0454 (15)	−0.0315 (15)	−0.0090 (13)	−0.0105 (14)
N1	0.0328 (12)	0.0258 (11)	0.0303 (12)	−0.0057 (9)	−0.0062 (10)	−0.0062 (9)
N2	0.0333 (12)	0.0278 (11)	0.0303 (12)	−0.0066 (10)	−0.0027 (9)	−0.0097 (9)
C1	0.0314 (15)	0.0322 (14)	0.0277 (13)	−0.0045 (11)	0.0009 (11)	−0.0051 (11)
C2	0.0330 (15)	0.0418 (16)	0.0318 (14)	−0.0096 (13)	0.0002 (12)	−0.0090 (12)
C3	0.0389 (17)	0.062 (2)	0.0282 (15)	−0.0089 (15)	−0.0055 (13)	−0.0022 (14)
C4	0.0456 (19)	0.054 (2)	0.0313 (16)	−0.0077 (16)	−0.0032 (14)	0.0097 (14)
C5	0.0410 (17)	0.0366 (16)	0.0398 (17)	−0.0095 (13)	0.0000 (13)	0.0027 (13)
C6	0.0343 (15)	0.0336 (14)	0.0312 (14)	−0.0069 (12)	−0.0003 (12)	−0.0027 (11)
C7	0.0381 (16)	0.0301 (14)	0.0351 (15)	−0.0122 (12)	−0.0017 (12)	−0.0044 (11)
C8	0.0363 (16)	0.0256 (13)	0.0457 (17)	−0.0011 (12)	−0.0074 (13)	−0.0077 (12)
C9	0.0366 (16)	0.0295 (14)	0.0437 (17)	−0.0018 (12)	−0.0030 (13)	−0.0106 (12)
C10	0.0375 (16)	0.0448 (17)	0.0349 (15)	−0.0111 (14)	−0.0095 (12)	−0.0070 (13)
C11	0.0340 (15)	0.0336 (14)	0.0382 (15)	−0.0090 (12)	−0.0086 (12)	−0.0081 (12)
C12	0.0452 (18)	0.0361 (15)	0.0311 (15)	−0.0103 (13)	0.0027 (13)	−0.0139 (12)
C13	0.0400 (16)	0.0378 (15)	0.0289 (14)	−0.0151 (13)	−0.0007 (12)	−0.0062 (12)
C14	0.054 (2)	0.0484 (19)	0.0301 (15)	−0.0218 (16)	−0.0024 (14)	−0.0067 (13)
C15	0.054 (2)	0.054 (2)	0.0309 (16)	−0.0227 (17)	−0.0102 (14)	0.0056 (14)
C16	0.0480 (19)	0.0357 (16)	0.0442 (18)	−0.0126 (14)	−0.0030 (15)	0.0044 (13)
C17	0.0409 (17)	0.0338 (15)	0.0357 (15)	−0.0146 (13)	0.0056 (13)	−0.0074 (12)
C18	0.0335 (15)	0.0340 (14)	0.0309 (14)	−0.0137 (12)	0.0010 (11)	−0.0054 (11)

### Geometric parameters (Å, °)

**Table:**

Cu1—O1	1.917 (2)	C12—C13	1.508 (4)
Cu1—O2	1.929 (2)	C13—C14	1.392 (4)
Cu1—O3	2.682 (3)	C13—C18	1.413 (4)
Cu1—N1	2.028 (2)	C14—C15	1.369 (5)
Cu1—N2	2.038 (2)	C15—C16	1.369 (5)
F1—C2	1.363 (4)	C16—C17	1.375 (4)
F2—C4	1.366 (4)	C17—C18	1.400 (4)
F3—C15	1.370 (3)	O3—H17	0.77 (5)
F4—C17	1.359 (3)	O3—H18	0.76 (6)
O1—C1	1.331 (3)	C3—H1	0.930
O2—C18	1.330 (3)	C5—H2	0.930
N1—C7	1.474 (3)	C7—H3	0.970
N1—C8	1.470 (3)	C7—H4	0.970
N1—C11	1.490 (4)	C8—H5	0.970
N2—C9	1.475 (3)	C8—H6	0.970
N2—C10	1.482 (4)	C9—H7	0.970
N2—C12	1.470 (4)	C9—H8	0.970
C1—C2	1.393 (4)	C10—H9	0.970
C1—C6	1.420 (4)	C10—H10	0.970
C2—C3	1.370 (4)	C11—H11	0.970
C3—C4	1.381 (5)	C11—H12	0.970
C4—C5	1.365 (5)	C12—H13	0.970
C5—C6	1.396 (4)	C12—H14	0.970
C6—C7	1.500 (4)	C14—H15	0.930
C8—C9	1.535 (4)	C16—H16	0.930
C10—C11	1.537 (4)		
			
O1—Cu1—O2	98.05 (9)	F3—C15—C16	118.9 (3)
O1—Cu1—N1	94.92 (9)	C14—C15—C16	122.1 (3)
O1—Cu1—O3	88.73 (10)	C15—C16—C17	117.1 (3)
O1—Cu1—N2	167.68 (9)	F4—C17—C16	117.9 (2)
O2—Cu1—N1	165.01 (9)	F4—C17—C18	117.3 (2)
O2—Cu1—N2	94.21 (9)	C16—C17—C18	124.8 (3)
O2—Cu1—O3	85.04 (10)	O2—C18—C13	124.6 (2)
O3—Cu1—N1	102.79 (10)	O2—C18—C17	120.0 (2)
O3—Cu1—N2	91.14 (10)	C13—C18—C17	115.3 (2)
N1—Cu1—N2	73.10 (10)	H17—O3—H18	108 (6)
Cu1—O1—C1	116.27 (17)	C2—C3—H1	121.6
Cu1—O2—C18	121.3 (2)	C4—C3—H1	121.6
Cu1—N1—C7	116.32 (19)	C4—C5—H2	120.1
Cu1—N1—C8	101.17 (19)	C6—C5—H2	120.1
Cu1—N1—C11	104.88 (17)	N1—C7—H3	109.2
C7—N1—C8	113.5 (2)	N1—C7—H4	109.2
C7—N1—C11	111.7 (2)	C6—C7—H3	109.2
C8—N1—C11	108.4 (2)	C6—C7—H4	109.2
Cu1—N2—C9	104.26 (18)	H3—C7—H4	107.9
Cu1—N2—C10	101.31 (17)	N1—C8—H5	110.3
Cu1—N2—C12	117.3 (2)	N1—C8—H6	110.3
C9—N2—C10	108.3 (2)	C9—C8—H5	110.3
C9—N2—C12	112.0 (2)	C9—C8—H6	110.3
C10—N2—C12	112.8 (2)	H5—C8—H6	108.6
O1—C1—C2	120.1 (2)	N2—C9—H7	110.2
O1—C1—C6	124.4 (2)	N2—C9—H8	110.2
C2—C1—C6	115.5 (2)	C8—C9—H7	110.2
F1—C2—C1	116.8 (2)	C8—C9—H8	110.2
F1—C2—C3	118.2 (3)	H7—C9—H8	108.5
C1—C2—C3	125.0 (3)	N2—C10—H9	110.3
C2—C3—C4	116.9 (3)	N2—C10—H10	110.3
F2—C4—C3	118.3 (3)	C11—C10—H9	110.3
F2—C4—C5	119.4 (3)	C11—C10—H10	110.3
C3—C4—C5	122.3 (3)	H9—C10—H10	108.5
C4—C5—C6	119.7 (3)	N1—C11—H11	110.3
C1—C6—C5	120.5 (3)	N1—C11—H12	110.3
C1—C6—C7	119.0 (2)	C10—C11—H11	110.3
C5—C6—C7	120.4 (3)	C10—C11—H12	110.3
N1—C7—C6	112.0 (2)	H11—C11—H12	108.5
N1—C8—C9	107.1 (2)	N2—C12—H13	108.8
N2—C9—C8	107.6 (2)	N2—C12—H14	108.8
N2—C10—C11	107.1 (2)	C13—C12—H13	108.8
N1—C11—C10	107.2 (2)	C13—C12—H14	108.8
N2—C12—C13	113.7 (2)	H13—C12—H14	107.7
C12—C13—C14	119.2 (2)	C13—C14—H15	120.0
C12—C13—C18	119.8 (2)	C15—C14—H15	120.0
C14—C13—C18	120.6 (2)	C15—C16—H16	121.5
C13—C14—C15	120.0 (3)	C17—C16—H16	121.5
F3—C15—C14	119.0 (3)		
			
O1—Cu1—O2—C18	149.2 (2)	C9—N2—C12—C13	167.7 (2)
O2—Cu1—O1—C1	147.0 (2)	C12—N2—C9—C8	−165.5 (2)
O1—Cu1—N1—C7	−4.8 (2)	C10—N2—C12—C13	−69.9 (3)
O1—Cu1—N1—C8	118.61 (18)	C12—N2—C10—C11	177.0 (2)
O1—Cu1—N1—C11	−128.73 (17)	O1—C1—C2—F1	−2.3 (4)
N1—Cu1—O1—C1	−40.5 (2)	O1—C1—C2—C3	178.0 (2)
O1—Cu1—N2—C9	40.3 (5)	O1—C1—C6—C5	−178.5 (2)
O1—Cu1—N2—C10	−72.0 (5)	O1—C1—C6—C7	5.0 (4)
O1—Cu1—N2—C12	164.8 (4)	C2—C1—C6—C5	3.2 (4)
N2—Cu1—O1—C1	−27.3 (6)	C2—C1—C6—C7	−173.3 (2)
O2—Cu1—N1—C7	145.0 (3)	C6—C1—C2—F1	176.1 (2)
O2—Cu1—N1—C8	−91.5 (3)	C6—C1—C2—C3	−3.6 (4)
O2—Cu1—N1—C11	21.1 (4)	F1—C2—C3—C4	−177.8 (2)
N1—Cu1—O2—C18	−0.4 (4)	C1—C2—C3—C4	1.9 (4)
O2—Cu1—N2—C9	−134.0 (2)	C2—C3—C4—F2	−179.6 (2)
O2—Cu1—N2—C10	113.62 (17)	C2—C3—C4—C5	0.5 (5)
O2—Cu1—N2—C12	−9.5 (2)	F2—C4—C5—C6	179.3 (2)
N2—Cu1—O2—C18	−32.0 (2)	C3—C4—C5—C6	−0.8 (5)
N1—Cu1—N2—C9	54.1 (2)	C4—C5—C6—C1	−1.2 (4)
N1—Cu1—N2—C10	−58.25 (17)	C4—C5—C6—C7	175.3 (2)
N1—Cu1—N2—C12	178.6 (2)	C1—C6—C7—N1	−55.8 (3)
N2—Cu1—N1—C7	178.1 (2)	C5—C6—C7—N1	127.6 (2)
N2—Cu1—N1—C8	−58.47 (18)	N1—C8—C9—N2	−8.7 (3)
N2—Cu1—N1—C11	54.19 (16)	N2—C10—C11—N1	−8.6 (3)
Cu1—O1—C1—C2	−135.5 (2)	N2—C12—C13—C14	133.7 (3)
Cu1—O1—C1—C6	46.3 (3)	N2—C12—C13—C18	−52.9 (4)
Cu1—O2—C18—C13	37.3 (4)	C12—C13—C14—C15	171.3 (3)
Cu1—O2—C18—C17	−144.8 (2)	C12—C13—C18—O2	7.7 (5)
Cu1—N1—C7—C6	47.3 (2)	C12—C13—C18—C17	−170.3 (3)
Cu1—N1—C8—C9	51.3 (2)	C14—C13—C18—O2	−179.0 (3)
Cu1—N1—C11—C10	−38.0 (2)	C14—C13—C18—C17	3.0 (4)
C7—N1—C8—C9	176.6 (2)	C18—C13—C14—C15	−2.0 (5)
C8—N1—C7—C6	−69.4 (3)	C13—C14—C15—F3	−179.9 (2)
C7—N1—C11—C10	−164.8 (2)	C13—C14—C15—C16	−0.1 (4)
C11—N1—C7—C6	167.7 (2)	F3—C15—C16—C17	−179.3 (3)
C8—N1—C11—C10	69.4 (2)	C14—C15—C16—C17	1.0 (5)
C11—N1—C8—C9	−58.7 (3)	C15—C16—C17—F4	−178.0 (3)
Cu1—N2—C9—C8	−37.7 (3)	C15—C16—C17—C18	0.3 (5)
Cu1—N2—C10—C11	50.8 (2)	F4—C17—C18—O2	−2.0 (4)
Cu1—N2—C12—C13	47.2 (3)	F4—C17—C18—C13	176.1 (2)
C9—N2—C10—C11	−58.5 (3)	C16—C17—C18—O2	179.6 (3)
C10—N2—C9—C8	69.6 (3)	C16—C17—C18—C13	−2.3 (5)

### Hydrogen-bond geometry (Å, °)

**Table:**

*D*—H···*A*	*D*—H	H···*A*	*D*···*A*	*D*—H···*A*
O3—H17···O2^i^	0.77 (5)	2.14 (5)	2.852 (4)	154 (5)
O3—H18···F1^i^	0.76 (6)	2.41 (6)	3.122 (3)	156 (6)
C7—H3···O2^ii^	0.97	2.49	3.376 (3)	152 (1)
C11—H12···O1^ii^	0.97	2.50	3.226 (3)	132 (1)
C8—H6···F4^iii^	0.97	2.54	3.356 (4)	143 (1)
C12—H13···F1^i^	0.97	2.32	3.117 (4)	140 (1)

Symmetry codes: (i) −*x*, −*y*, −*z*+1; (ii) −*x*+1, −*y*, −*z*+1; (iii) *x*, *y*+1, *z*.

## References

[bb1] Altomare, A., Cascarano, G., Giacovazzo, C. & Guagliardi, A. (1993). *J. Appl. Cryst.* **26**, 343–350.

[bb2] Bernstein, J., Davis, R. E., Shimoni, L. & Chang, N.-L. (1995). *Angew. Chem. Int. Ed. Engl.* **34**, 1555–1573.

[bb3] Kubono, K., Hirayama, N., Kokusen, H. & Yokoi, K. (2003). *Anal. Sci.* **19**, 645–646. 10.2116/analsci.19.645 12725412

[bb4] Kubono, K., Noshita, C., Tani, K. & Yokoi, K. (2009). *Acta Cryst.* E**65**, m1685–m1686. 10.1107/S1600536809049800 PMC2971750 21578691

[bb5] Loukiala, S., Ratilainen, J., Valkonen, J. & Rissanen, K. (1997). *Acta Chem. Scand.* **51**, 1162–1168.

[bb6] Mukhopadhyay, S., Mandal, D., Chatterjee, P. B., Desplanches, C., Sutter, J.-P., Butcher, R. J. & Chaudhury, M. (2004). *Inorg. Chem.* **43**, 8501–8509. 10.1021/ic049271r 15606199

[bb7] North, A. C. T., Phillips, D. C. & Mathews, F. S. (1968). *Acta Cryst.* A**24**, 351–359.

[bb8] Rigaku/MSC (2006). *WinAFC* and *CrystalStructure* Rigaku/MSC, The Woodlands, Texas, USA.

[bb9] Sheldrick, G. M. (2008). *Acta Cryst.* A**64**, 112–122. 10.1107/S0108767307043930 18156677

[bb10] Spek, A. L. (2009). *Acta Cryst.* D**65**, 148–155. 10.1107/S090744490804362X PMC2631630 19171970

[bb11] Tsai, H.-A., Hu, M.-S., Teng, M.-Y., Suen, M.-C. & Wang, J.-C. (2008). *Polyhedron*, **27**, 2035–2042.

[bb12] Weinberger, P., Costisor, O., Tudose, R., Baumgartner, O. & Linert, W. (2000). *J. Mol. Struct.* **519**, 21–31.

[bb13] Zhao, X.-J., Du, M., Wang, Y. & Bu, X.-H. (2004). *J. Mol. Struct.* **692**, 155–161.

